# Drug development for exceptionally rare metabolic diseases: challenging but not impossible

**DOI:** 10.1186/1750-1172-8-179

**Published:** 2013-11-15

**Authors:** Michelle Putzeist, Aukje K Mantel-Teeuwisse, Christine C Gispen-de Wied, Arno W Hoes, Hubert GM Leufkens, Remco LA de Vrueh

**Affiliations:** 1 Utrecht Institute for Pharmaceutical Sciences, Division of Pharmacoepidemiology and Clinical Pharmacology, Utrecht University, P.O. Box 80 082, 3508TB Utrecht, The Netherlands; 2 Medicines Evaluation Board, Utrecht, The Netherlands; 3 Julius Center for Health Sciences and Primary Care, University Medical Center Utrecht, Utrecht, The Netherlands; 4 Rare Disease Matters, Leiden, The Netherlands

**Keywords:** Rare disease, Orphan medicinal product, Inherited metabolic disease, Prevalence, Preclinical proof of concept

## Abstract

**Background:**

We studied to what extent the level of scientific knowledge on exceptionally rare metabolic inherited diseases and their potential orphan medicinal products is associated with sponsors deciding to apply for an orphan designation at the US Food and Drug Administration (FDA) or the European Medicines Agency (EMA).

**Methods:**

All metabolic diseases with a genetic cause and prevalence of less than 10 patients per 1 million of the population were selected from the ‘Orphanet database of Rare diseases’. The outcome of interest was the application for an orphan designation at FDA or EMA. The level of publicly available knowledge of the disease and drug candidate before an orphan designation application was defined as whether the physiological function corresponding with the pathologic gene and initiation of the pathophysiological pathway was known, whether an appropriate animal study was identified for the disease, whether preclinical proof of concept was ascertained and the availability of data in humans. Other determinants included in the study were metabolic disease class, the prevalence of the disease, prognosis and time of first description of the disease in the literature. Univariate relative risks (RRs) and 95% confidence intervals (CIs) of an orphan designation application were calculated for each of these determinants. In addition, a multivariate Cox regression analysis was conducted (Forward LR).

**Results:**

In total, 166 rare metabolic genetic diseases were identified and included in the analysis. For only 42 (25%) of the diseases an orphan designation application was submitted at either FDA or EMA before January 2012. The multivariate analysis identified preclinical proof of concept of a potential medicinal product as major knowledge related determinant associated with an orphan designation application (RRadj 3.9, 95% CI 1.9-8.3) and confirmed that prevalence of the disease is also associated with filing an application for an orphan designation (RRadj 2.8, 95% CI 1.4-5.4).

**Conclusion:**

For only one out of four known exceptionally rare metabolic inherited diseases sponsors applied for an orphan designation at FDA or EMA. These applications were found to be associated with the prevalence of the rare disease and the level of available scientific knowledge on the proof of concept linking possible drug candidates to the disease of interest.

## Introduction

Rare diseases are a complex and heterogeneous mosaic of an estimated 6000–8000 conditions. Several jurisdictions, including the US and the EU, have successfully introduced specific legislation with a number of (economic) incentives (see Table 1) that stimulate the development of products for rare diseases [1-4]. In the first 25 years of the Orphan Drug Act in the US 1892 products have been designated as orphan, and 326 products have been approved [5]. These 326 products target more than 200 rare diseases and represent a difference in the lives of millions of rare disease patients. In the EU, in the first decade more than 850 orphan drug designations have been granted by the European Commission and more than 60 orphan drugs have received marketing authorization [6].

**Table 1:** Orphan designation criteria at EMA and FDA

**Orphan designation criteria**	
European Medicines Agency	Food and Drug Administration
**(i)** The medicine should be intended for the diagnosis, prevention or treatment of a life-threatening or chronically debilitating disease, affecting a maximum of 500 in 1 million people in the EU;	**(i)** The medicine should be intended for a disease or condition that affects fewer than 200,000 people in the United States or, if the drug is a vaccine, diagnostic drug, or preventive drug, the persons to whom the drug will be administered in the United States are fewer than 200,000 per year;
**(ii)** It must be unlikely that the revenue after marketing authorisation will cover the investments in its development;	**(ii)** There is no reasonable expectation that costs of research and development of the drug for the indication can be recovered by sales of the drug in the United States;
**(iii)** No satisfactory treatment for the disease exists or the new medicinal product is of significant benefit to the patients;	**(iii)** An application for an orphan designation should contain the reasons why such therapy is needed, accompanied by a discussion of the scientific rationale for the use of the drug for the rare disease, including all data from nonclinical laboratory studies, clinical investigations, and other relevant data that are available to the sponsor, whether positive, negative, or inconclusive. Copies of pertinent unpublished and published papers are also required [4].
**(iv)** An application for an orphan designation should explain the medical rationale of the medicinal product by means of the mechanism of action as far as it’s known, and some preclinical or clinical date are ‘generally’ required, including all published references [3].	

Despite this success, it is important to understand that the majority of the estimated 6000–8000 rare diseases has a prevalence of less than 10 patients per 1 million inhabitants (less than 5000 patients in the EU) [7]. The risk exists that such a small number of patients and consequently small market size make it less attractive for the pharmaceutical industry to invest in the development of therapies for low prevalence rare diseases. Heemstra et al. and Yin et al. showed that translation of rare disease research into an orphan drug development program is more likely for a more prevalent rare disease than a less prevalent rare disease [8,9]. However, a recent overview by the FDA clearly revealed that orphan medicinal products approved for low prevalence rare diseases are certainly not uncommon [5]. An example of an authorized product to treat a low prevalence rare metabolic disease is idursulfase (Elaprase®), an enzyme replacement therapy to treat Hunter syndrome also known as mucopolysaccharidosis Type II [10]. Hunter syndrome is a very rare genetic life-threatening lysosomal storage disease characterized by the accumulation of glycosaminoglycans due to the deficiency of the enzyme iduronate-2-sulfatase [10,11]. Apart from idursulfase, other marketed products for low prevalence rare metabolic diseases are for example nitisinone (Orfadin® for Tyrosinemia type I) and carglumic acid (Carbaglu® for N -acetylglutamate synthetase (NAGS) deficiency).

Apparently, drug development for low prevalence rare disorders may be less obvious, perhaps challenging, but certainly not impossible. Considering that the majority of low prevalence rare diseases remain without therapy [6,8], increased knowledge of the underlying translational process provides better input for novel approaches to improve orphan drug development. Heemstra et al. showed that disease-specific scientific output was a predictive factor for successful translation of rare disease research into an orphan drug development program [8]. However, the authors did not differentiate between different research areas such as disease etiology and pathophysiology, availability of suitable animal models and/or (pre-) clinical proof of concept studies. Therefore, we studied to what extent disease characteristics as well as the level of publicly available scientific knowledge on low prevalence rare metabolic diseases and its potential medicinal products is associated with a sponsor’s decision to apply for an orphan designation at FDA or EMA.

## Methods

All rare metabolic diseases (inborn errors of metabolism) were downloaded from the ‘Orphanet database of Rare diseases’ on 17 January 2012 [12]. Subsequently, diseases with a prevalence of less than 10 patients per 1 million of the population and for which a genetic cause has been established (heritance known) were extracted from the dataset and included in the study (N = 166). The outcome of interest for this study was the first orphan designation application at the FDA or EMA. In case of multiple orphan designation applications for the same rare disease indication at either FDA and/or EMA, we selected the first application as a proxy for the intention to initiate the development of a drug for the rare disease leading to marketing authorization [13,14].

Determinants that described the level of available scientific knowledge of the disease before the first orphan designation application were: (1) whether the gene function corresponding with the pathologic gene and initiation of the pathophysiological pathway was identified (yes, no) and (2) whether an appropriate animal model was available for the disease (yes, no). Scientific knowledge related to drug candidates was (3) preclinical proof of concept of any drug candidate either in vitro or in an animal model of the disease and (4) the availability of data in humans. The availability of data in humans was defined as any clinical testing of a drug candidate in patients with the rare disease, irrespective of the type of treatment (symptomatic or curative), the underlying study (a case report or a comparative study), whether the treatment was successful or not, and - in case of the diseases for which an orphan designation was available- irrespective of whether the drug described was the drug of the orphan designation application.

Data about the gene function and proteins or organelles involved in initiating the pathophysiological pathway and the availability of an appropriate animal model were identified from the OMIM database of genetic diseases [15]. Pubmed publications were the data source for the animal model (additional to OMIM), preclinical proof of concept and the availability of clinical data. All Pubmed publications for each disease were identified by Pubmed search strings according to Heemstra et al. [8], taking into account all available synonyms for the disease and the date of the orphan designation application (for diseases with an orphan designation) or the cut-off date 01-01-2012 (for the diseases without orphan designation) [16]. The availability of clinical studies was verified at clinicaltrial.gov [17].

Other determinants that were studied were the metabolic disease class according to the Orphanet classification, the prevalence of the disease (<1 per 1 million of population or 1–9 per 1 million of population) and the prognosis of the disease (fatal/chronically debilitating or non fatal/not chronically debilitating). Besides, the period in which the disease was first described (before 1977 or starting from 1977) was assessed to study the association with time.

The disease class, prognosis and prevalence were all collected from Orphanet, as indicated in July 2012 [18]. In case of multiple prognoses depending on disease severity, the worst prognosis was included. The year in which the disease was first described in the scientific literature was derived from OMIM [17]. For ten diseases OMIM did not mention the year the disease was first described, and consequently another public source of information (Pubmed and other public references) was used to retrieve the data.

Univariate relative risks (RRs) of applying for an orphan designation and 95% confidence intervals (CIs) were calculated for each of these determinants. In addition, a multivariate Cox regression analysis was conducted (Forward LR) to obtain adjusted relative risks (RRadj). All analyses were done using SPSS, version 19.0. The most recent year that a rare inherited metabolic disease with an orphan designation was first described was 1997. Absence of an orphan designation for diseases first described after 1997 may be due to insufficient time to translate fundamental disease knowledge into sufficient (pre-)clinical data required for the application of an orphan designation. Therefore, a sensitivity analysis was performed in which all diseases that were first described after 1997 were excluded.

## Results

In total 166 metabolic genetic diseases with a prevalence of less than 10 per 1 million patients were identified from the Orphanet database of Rare diseases. Table 2 provides the Orphanet classification of these diseases [18]. This table shows that three metabolic disease subclasses, i.e. lysosomal diseases (subclass of Metabolic diseases involving complex molecules), protein metabolism disorders (subclass of Metabolic Intoxication diseases) and mitochondrial disorders (subclass of Energy metabolism disorders) represented more than half of the study diseases (N = 91). The other inherited metabolic diseases were a heterogenous group of disorders.

**Table 2:** Classification of exceptionally rare diseases included in the present study according to Orphanet

**General classification**	**Total (N = 166)**	**Disease group classification**	**Total**
Metabolic disease involving complex molecules	62	Lysosomal diseases	31 (50%)
		Purine or pyrimidine metabolism disorder	10 (16%)
		Sterol metabolism disorder	8 (13%)
		Metabolic neurotransmission anomaly	5 (8%)
		Peroxisomal disease	4 (6%)
		Metal transport or utilisation disorder	3 (5%)
		Protein glycosylation disorder	1 (2%)
Metabolic intoxication disease	36	Amino or protein metabolism disorder	34 (94%)
		Hyperoxaluria	1 (3%)
		Methylmalonic aciduria - microcephaly - cataract	1 (3%)
Energy metabolism disorder	45	Mitochondrial disorder	26 (58%)
		Fatty acid oxidation or ketogenesis disorder	7 (9%)
		Creatine biosynthesis disorder	2 (4%)
		Ketolysis disorder	2 (4%)
		Enolase deficiency	1 (2%)
		Gluconeogenesis disorder	1 (2%)
		Hemolytic anemia due to glucophosphate isomerase deficiency	1 (2%)
		Phosphoglycerate kinase 1 deficiency	1 (2%)
		Pyruvate metabolism disorder	1 (2%)
		Thiamine-responsive megaloblastic anemia syndrome	1 (2%)
		Tricarboxylic acid cycle disorder	1 (2%)
		Triose phosphate-isomerase deficiency	1 (2%)
Carbohydrate metabolism disorder	12	Glycogen storage disease	7 (58%)
		Glucose transport disorder	4 (33%)
		Familial hyperinsulinism	1 (8%)
Other metabolic disease	11	Metabolic disease associated with a progressive neurological disorder	6 (55%)
		Miscellanous metabolic disease with mostly hepatic presentation	4 (36%)
		Hereditary hypercarotenemia and vitamin A deficiency	1 (9%)

**Figure 1:**
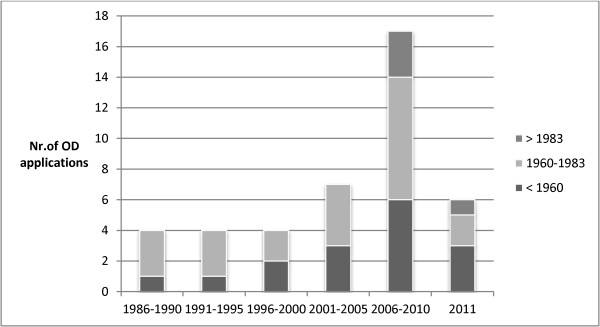
Number of first applications for an orphan designation at FDA or EMA over time and according to the period in which the exceptionally rare metabolic inherited diseases were first described.

For 42 (25%) of the diseases at least one orphan designation application was submitted at either FDA or EMA, whereas for the remaining 124 (75%) diseases such an orphan designation application was not submitted before January 2012. Figure 1 depicts the 42 orphan designations applications over time. After 2000 there was an increase in the number of orphan designation applications which became even larger from 2006 onwards. The figure also shows when low prevalence rare metabolic diseases with an orphan designation were first described in the scientific literature. Diseases with a long history, first described in the scientific literature before 1960, were still well represented among the orphan designation applications in 2011. First orphan designation applications for relatively new diseases, described after 1983, were submitted from 2006 onwards. A bibliometric analysis of the 166 low prevalence rare metabolic diseases showed a clear difference between the group of low prevalence rare metabolic diseases with an orphan designation and the group of diseases without an orphan designation. More than 50% (N = 23) of the 42 diseases with an orphan designation application had a scientific output of more than 200 scientific papers. By contrast, only 9% (N = 11) of the 124 diseases without an orphan designation application had a scientific output of more than 200 scientific papers.

Table 3 demonstrates the univariate relative risks (RR) of submitting an orphan designation application at FDA or EMA for different disease characteristics and type/level of publicly available scientific knowledge. Considering the level of scientific knowledge preclinical proof of concept of drug candidates had the largest univariate RR of an orphan designation application (RR 6.0 (95% CI 3.0-12.0)). The availability of data in humans (RR 3.3 (95% CI 1.7-6.6)) and the availability of an animal model (RR 3.0 (95% CI 1.5-6.0)) also demonstrated a positive association with the likelihood that a sponsor had filed an orphan designation application. A similar, but not significant, association was found for the identification of the gene function and the underlying cause of the disease (RR 26.1 (95% CI 0.7-966)). For none of the 26 diseases for which the gene function was not yet fully identified an orphan designation application was submitted. Ten out of these 26 diseases were mitochondrial diseases (subclass of energy metabolism disorders), which suggests that for this disease group the pathophysiological pathway requires further elucidation.

**Table 3:** Univariate relative risks of an orphan designation application at EMA or FDA for different disease characteristics and for publicly available scientific knowledge

**Disease characteristics**	**Total N = 166**	**OD application N = 42**	**No OD application N = 124**	**Univariate RR (95% CI)**
**1. Metabolic disease class**				
Metabolic disease involving complex molecules	62	23 (37%)	39 (63%)	Ref
Metabolic intoxication disease	36	14 (38%)	22 (61%)	1.1 (0.5–2.0)
Energy metabolism disorder	45	3 (7%)	42 (93%)	0.2 (0.1–0.6)
Carbohydrate metabolism disorder	12	1 (8%)	11 (92%)	0.2 (0.03–1.7)
Other metabolic disease	11	1 (9%)	10 (91%)	0.3 (0.03–1.8)
**2. First description of the disease**				
≤ 1977	98	36 (37%)	62 (63%)	4.2 (1.8–9.9)
> 1977	68	6 (9%)	62 (91%)	Ref
**3. Prevalence**				
1-9/1.000.000	38	25 (66%)	13 (34%)	5.0 (2.7–9.2)
<1/1.000.000	128	17 (13%)	111(87%)	Ref
**4. Prognosis**				
Fatal/chronically debilitating despite treatment	96	34 (35%)	62 (65%)	2.7 (1.3–5.8)
Non fatal/not chronically debilitating	61	8 (13%)	53 (87%)	Ref
Unknown	9	0 (0%)	9 (100%)	NA
**Scientific knowledge related variables**				
**5. Gene function identified?**				
Yes	140	42 (30%)	98 (70%)	26.1 (0.7–966)
No	26	0 (0%)	26(100%)	Ref
**6. Animal model available?**				
Yes	87	33 (38%)	54 (62%)	3.0 (1.5–6.0)
No	79	9 (11%)	70 (89%)	Ref
**7. Preclinical proof of concept?**				
Yes	53	31 (58%)	22 (42%)	6.0 (3.0–12.0)
No	113	11 (10%)	102(90%)	Ref
**8. In humans data available?**				
Yes	76	31 (40%)	45 (59%)	3.3 (1.7–6.6)
No	90	11 (12%	79 (88%)	Ref

The metabolic diseases involving complex molecules and the metabolic intoxication diseases were similarly associated with orphan designation applications, whereas the energy and carbohydrate metabolism disorders were less likely to have an orphan designation application (RR = 0.2 (95% CI 0.1-0.6) as compared to metabolic diseases involving complex molecules). The positive associations with an orphan designation application for metabolic diseases involving complex molecules and metabolic intoxication diseases were mainly driven by lysosomal (storage) diseases and amino or protein metabolism disorders, respectively. For 17 of 31 (55%) lysosomal diseases and for 13 of 34 (38%) of amino or protein metabolism disorders an orphan designation application was submitted. Finally, a relatively high prevalence (1–9 per 1 million), poor disease prognosis and first description of the disease ≤1977 were all positively associated with an orphan designation application (Table 3).

The multivariate analysis (Table 4) confirmed a strong association between the preclinical proof of concept of potential medicinal products and applying for an orphan designation (RRadj 3.9 95% CI 1.9-8.3) and also proved that prevalence of the disease is associated with an orphan designation application (RRadj2.8 95% CI 1.4-5.4). An additional sensitivity analysis that excluded all diseases that were first described after 1997 demonstrated only small differences in (adjusted) RRs (data not shown).

**Table 4:** Multivariate relative risks of an application for an orphan designation at EMA or FDA

**Variable**	**Univariate RR (95% CI)**	**Multivariate RR (95% CI)**
**Prevalence**		
1-9/1.000.000	5.0 (2.7–9.2)	2.8 (1.4–5.4)
<1/1.000.000	Ref	Ref
**Preclinical proof of concept?**		
Yes	6.0 (3.0–12.0)	3.9 (1.9–8.3)
No	Ref	Ref

## Discussion

The majority of low prevalence rare diseases remain without therapy, the development of medicines for such diseases is considered an unmet medical need. This study demonstrates the importance of mature scientific knowledge in the public domain for successful translation of rare disease research into an orphan drug development program, in line with Heemstra et al. [8]. The preclinical proof of concept of a drug candidate and disease prevalence were identified as important factors driving sponsors to apply for an orphan designation for a drug candidate. The orphan designation application rather than designation approval was taken as study outcome, because we were interested in the disease characteristics and level of disease knowledge available at time of the initiative of drug development for low prevalence rare metabolic diseases. In practice all but one designation applications were approved. Thus, our study also demonstrates the association between the level of knowledge of the disease and/or drug and granting orphan designations.

### Knowledge of the disease

Our results demonstrate that for the majority of low prevalence rare metabolic diseases included in the study the causative gene and initiation of the pathophysiological pathway was known and that the diseases for which this knowledge was lacking belonged to similar metabolic disease subclasses (e.g. mitochondrial diseases). Although basic knowledge of the disease is generally considered a prerequisite for further (pre)clinical drug development in general [19,20], the elucidation of the gene function was not identified as an independent driver for sponsors to apply for an orphan designation. A total of 98 (70%) low prevalence rare genetic metabolic diseases with an elucidated gene function did not have an orphan designation. Apparently, additional and more important reasons exist that explain the observed lack of orphan drug development for low prevalence rare metabolic diseases. First, elucidation of the gene function is only one component of the elucidation of the complete pathophysiological pathway from gene (translation) to clinical symptoms and the identification of druggable targets. Secondly, even if extensive disease knowledge is available this does not guarantee the successful development of an orphan drug. For example, cystic fibrosis, an inherited chronic disease that affects 70,000 people worldwide, has been studied extensively and the pathophysiology is well known. However, the first medicine to treat the underlying cause of cystic fibrosis, ivacaftor (Kalydeco®) was only approved recently [21].

Still, pathophysiological knowledge of one disease within a disease subclass may stimulate or act as catalyst for disease research for other diseases within the same subclass. For the mucopolysaccharidosis diseases (5 out of 7 with an orphan designation) the underlying pathophysiology is relatively well understood: a lack of specific lysosomal enzymes leads to the degradation of glycosaminoglycans or mucopolysaccharides. The accumulation of partially degraded glycosaminoglycans causes interference with cell, tissue, and organ function causing severe clinical symptoms [11]. Just like in the example of idursulfase to treat Hunter syndrome or mucopolysaccharidosis Type II, enzyme replacement therapies have been developed as a response to enzyme deficiencies in several other mucopolysaccharidosis diseases. Similarly within the group of amino or protein metabolism disorders all five urea cycle disorders, leading to ammonia detoxification, received an orphan designation. The urea cycle is well studied and several treatment strategies have been developed such as drug suppletion therapies (arginine therapy) or a druggable target such as circulating nitrogen (designated product glyceryl tri-(4-phenylbutyrate).

In addition, there is circumstantial evidence which suggests that availability of a (potential) therapeutic modality for a specific rare disease appears to encourage rather than discourage additional orphan drug development for the same rare disorder [22]. As such crossing the translational chasm between research and development not only represents the development of a potential life-saving or quality of life-improving therapy for rare disease patients, but may also have an important spin-off effect. For example, rare diseases share (parts of) the same biochemical pathway, and consequently an orphan drug may be beneficial for more than one disease. Nitisinone, a product approved for tyrosinemia type I (Orfadin®) may also potentially have a beneficial clinical effect for patients suffering from alkaptonuria (AKU). The AKU society, a patient association, is currently collaborating with academia and industry to study the potential clinical effect of nitisinone for AKU [23].

These examples underline that the availability of disease-related knowledge in the public domain is crucial to initiate an orphan drug development program by pharmaceutical companies, but also increasingly by disease-focused foundations [24].

### Knowledge of the drug

Preclinical proof of concept of drug candidates was identified as the major type of knowledge needed for an orphan designation. According to our definition, preclinical proof of concept was considered achieved in case of a promising result in an *in vitro* or animal study with any drug candidate studied in the target population, irrespective of the drug that was included in the orphan designation application. To obtain an orphan designation a sponsor has to provide (pre-)clinical data that confirm the medical plausibility of the intended drug candidate [3,4]. Yet, for 40% of the metabolic diseases for which proof of concept was demonstrated in a preclinical study, an orphan designation was not applied for. Possible explanation related to low prevalence are the difficulties a sponsor may face during the subsequent clinical development because of the extremely small patient populations available for clinical research. Kakkis et al. recently identified 15 inherited exceptionally rare metabolic diseases with a relevant corresponding animal model, a treatment with a known mode of action and a clinically relevant treatment effect in animals but that had stalled in clinical development. They demonstrated the substantial potential benefit that surrogate endpoints could offer to clinical drug development. The acceptance of surrogate endpoints in clinical development of these promising treatments would reduce the number of patients needed for approval and may also persuade sponsors to apply for an orphan designation [25].

In contrast to preclinical proof of concept, clinical testing (data in humans) was only associated with an application for an orphan designation in the univariate analysis. This may be because of the way ‘data in humans’ were defined: as any treatment described in the scientific literature, (i) either successful or not, (ii) either a case report or a small trial and (iii) either a symptomatic or a curative treatment. The thought behind this definition was that (ad i) any drug developer could learn from published data about any drug treatment for the disease, (ad ii) that requiring a clinical trial was not realistic for low prevalence-rare diseases, and (ad iii) that orphan designations can be applied for symptomatic treatments as well as curative treatments.

### Other drivers for an orphan designation application

The dataset consisted of low prevalence rare metabolic diseases (<10/million), the majority (N = 128; 77%) of the diseases had a prevalence of less than 1 patient per million inhabitants. Our study showed that diseases with a prevalence of 1–9 per million had a higher chance of an orphan designation application than diseases with a prevalence of less than 1 per million. Our finding that prevalence is an important factor that drives sponsors to apply for an orphan designation is in line with previous results by Heemstra et al. [8]. For some diseases less than 20 cases have been described worldwide. Therefore, a likely explanation for this finding is that a disease prevalence of less than 1 per million is considered too small by the pharmaceutical industry to invest in the development of a therapy, despite the availability of considerable disease knowledge, incentives offered by the Orphan Regulation and opportunities for conditional marketing approval and approval under exceptional circumstances in the EU [26,27]. The latter is best illustrated by the group of gamma-glutamyl cycle disorders (N = 4, subclass of protein metabolism disorders), involved with the synthesis and degradation of glutathione. At the time of our analysis only about ten patients were described. Despite elucidation of the gamma-glutamyl cycle, the genes and proteins involved and the availability of knock-out mouse models no orphan designations have been applied for by sponsors at the time of our analysis.

Another explanation may be a lack of need of pharmacological treatment for some diseases, because either the symptoms are not severe (e.g. hereditary hypercarotenemia and vitamin A deficiency) or because other non-pharmacological treatments or life style changes are sufficient, such as diet or exercise restrictions (e.g. for glucose-galactose malabsorption) [28]. Third, medicinal products that are already approved to the market can be prescribed off-label to treat symptoms, e.g. anti-epileptics to treat convulsions as a consequence of progressive neuronal damage of McLeod neuroacanthocytosis syndrome [29] or vitamin B12 injections for cobalamine deficiency disorders such as Gräsbeck-Imerslund disease [30]. For these congenital disorders the focus may be on care rather than on cure. Finally, some metabolic diseases may be well treated by suppletion of amino acids such as arginine or carnitine (for some types of organic aciduria) or carbohydrates such as glucose (for ketolysis disorders) [18].

### Limitations and further research

The methodology of our study has some limitations, mainly related to potential discrepancies between the availability of knowledge in scientific literature and the knowledge referred to in the applications for orphan designation. We studied whether the knowledge as available in scientific literature was an incentive to initiate further research to the disease and drug candidates. However, an orphan designation application can also be based on unpublished studies, e.g. a preclinical proof of concept study of the drug candidate only known to the company or institution filing the application, but this information is not available in the public domain and could therefore not be included in the present study.

A limitation of this study is that the results do not identify whether the drug described in a publication of preclinical proof of concept or in a case report was similar to the drug included in the orphan designation application. This approach was needed to study both rare diseases with and without an application for an orphan designation in a similar way. This limitation could be considered acceptable, with the assumption that drug developers can learn from any experience with drug candidates targeting low prevalence rare diseases. Besides, the quality of the scientific output by disease was not taken into account. Our study demonstrated that for diseases first described before 1977 more orphan designations have been requested than for diseases that were first described after 1977. The quality of the scientific output as well as the repetition of results may play a role here and could be the subject of a follow-up study. Finally, we have to note that due to the small sample size in this study confidence intervals of relative risks were wide. Nevertheless, the relative risks and their confidence intervals derived from the (multivariate) regression analyses do identify the most relevant determinants of the application of an orphan designation.

### Overall conclusion and policy implications

The majority of low prevalence rare diseases remain without therapy. This study shows that for low prevalence rare metabolic diseases preclinical proof of concept of drug candidates and disease prevalence play an important role in the translation of disease knowledge into an orphan drug development program. To effectively expand drug development for low prevalence rare diseases we recommend that incentives by public funders and disease-focused foundations should (continue to) aim at stimulating fundamental research to elucidate the pathophysiology of the disease as well as the identification of druggable targets and the testing of potential drug candidates in a suitable preclinical model. Although not exclusively, the latter represents an important incentive for public or private partners to move a potential drug candidate into the clinical development stage. More importantly, an important hurdle will have been addressed towards the ultimate goal: a therapy for a patient suffering from a (low prevalence) rare disease.

## Competing interests

This study was funded by the Dutch Top Institute Pharma Escher project (T6-202). The WHO Collaborating Centre receives no direct funding or donations from private parties, including pharma industry. Research funding from public-private partnerships, e.g. IMI, TI Pharma (http://www.tipharma.nl) is accepted under the condition that no company-specific product or company related study is conducted. The Centre has received unrestricted research funding from public sources, e.g. Netherlands Organisation for Health Research and Development (ZonMw), the Dutch Health Care Insurance Board (CVZ), EU 7th Framework Program (FP7), Dutch Medicines Evaluation Board (MEB), and Dutch Ministry of Health. Remco de Vrueh is since 1-9-2013 employed as program manager at TI Pharma. The authors declare that they have no competing interests.

## Authors’ contributions

MP: wrote the research proposal, collected the data and conducted the data analysis. Subsequently she wrote the draft of the manuscript. AKMT: gave advice about the research proposal, data analysis and interpretation of the results. AKMT also revised the manuscript. HGML: gave advice about the research proposal and revised the manuscript. AWH: gave advice about data analysis and interpretation of the results and revised the manuscript. CCGDW: gave advice about the interpretation of the results and revised the manuscript. RLADV: initiated and partly wrote the research proposal, initiated the data collection and gave feedback on further data collection by MP. In addition he revised the manuscript. All authors read and approved the final manuscript.
